# Advances in dendritic cell targeting nano-delivery systems for induction of immune tolerance

**DOI:** 10.3389/fbioe.2023.1242126

**Published:** 2023-10-09

**Authors:** Guojiao Lin, Jialiang Wang, Yong-Guang Yang, Yuning Zhang, Tianmeng Sun

**Affiliations:** ^1^ Key Laboratory of Organ Regeneration and Transplantation of Ministry of Education, Institute of Immunology, The First Hospital, Jilin University, Changchun, China; ^2^ National-local Joint Engineering Laboratory of Animal Models for Human Diseases, Changchun, China; ^3^ International Center of Future Science, Jilin University, Changchun, China; ^4^ State Key Laboratory of Supramolecular Structure and Materials, Jilin University, Changchun, China

**Keywords:** dendritic cell, nanoparticles, immune tolerance, autoimmune diseases, transplantation, allergy

## Abstract

Dendritic cells (DCs) are the major specialized antigen-presenting cells (APCs), play a key role in initiating the body’s immune response, maintain the balance of immunity. DCs can also induce immune tolerance by rendering effector T cells absent and anergy, and promoting the expansion of regulatory T cells. Induction of tolerogenic DCs has been proved to be a promising strategy for the treatment of autoimmune diseases, organ transplantation, and allergic diseases by various laboratory researches and clinical trials. The development of nano-delivery systems has led to advances *in situ* modulation of the tolerance phenotype of DCs. By changing the material composition, particle size, zeta-potential, and surface modification of nanoparticles, nanoparticles can be used for the therapeutic payloads targeted delivery to DCs, endowing them with great potential in the induction of immune tolerance. This paper reviews how nano-delivery systems can be modulated for targeted delivery to DCs and induce immune tolerance and reviews their potential in the treatment of autoimmune diseases, organ transplantation, and allergic diseases.

## 1 Introduction

DCs were discovered in the early 1970s, since then their biological properties have been extensively studied. To date, they are known as the “gatekeepers of the immune system” due to their ability to maintain immune homeostasis by activating adaptive immunity or promoting tolerance (Steinman and Cohn, 1973; Steinman and Cohn, 1974; Puhr et al., 2015). DCs serve as immune sentinels, specifically responsible for sensing danger signals as well as capturing, processing, and presenting antigenic substances (Banchereau and Steinman, 1998). DCs can exert regulatory effects on T cells, controlling T cell activation, differentiation and expansion. Among the various phenotypes of DCs, tolerogenic DCs (tol-DCs) can induce T-cell tolerance and inhibit abnormal activation of the immune system through a variety of mechanisms (Tang et al., 2022). Animal models and preclinical studies have revealed that inducing immune tolerance using tol-DCs demonstrated therapeutic effects in autoimmune diseases, allergic diseases, and organ transplant-related diseases (Ness et al., 2021).

Tol-DCs can be induced by alterations in the physiological environment, and the development of nanodrug delivery systems provides an efficient and simple solution for the *in situ* induction of tol-DCs *in vivo* (Carey et al., 2023; Rui et al., 2023). Nano-delivery systems, which can deliver therapeutic agents to specific targets and reduce the side effects of drugs, have led to significant advances in the development of new imaging agents, disease therapies and biological tools such as genome editors and nanomachines (Poon et al., 2020). Nanodrug carriers can not only deliver immunosuppressive drugs, but also self-antigen-related peptides and nucleic acids, or in combinations. The engineered nanoparticles enable the targeted delivery of therapeutic cargoes to DCs and induces the generation of DCs with a tolerogenic phenotype to regulate antigen-specific immune responses *in vivo*.

Here, we review how the optimization of the physicochemical properties of DC targeting nano-delivery systems can improve the ability of nanoparticles to induce the tol-DC phenotype, as well as their therapeutic potential towards autoimmune diseases, allergy, and organ transplantation.

## 2 The role of immune tolerance

The most important function of the immune system is to recognize and eliminate invading antigens and malignant cells while maintaining immune tolerance to its own components. However, unrestricted immune system activation can lead to clinical disorders, including autoimmune diseases, solid organ transplantation (SOT), hematopoietic stem cell transplantation (HSCT) and allergic diseases (Choi et al., 2020; Zhuang et al., 2021; Chang et al., 2022). By inducing immune tolerance, that is, by inducing specific tolerances to disease-inducing immune cells, the body can avoid inflammation while retaining its normal immune response to foreign substances (Ezekian et al., 2018).

Immune tolerance arises from the control of self-reactive T cells in the thymus and periphery, known as central immune tolerance and peripheral immune tolerance, respectively (ElTanbouly and Noelle, 2021). During the positive selection of T cell development in thymus, T cells that recognizing own major histocompatibility complex (MHC) molecules are remained; while T cells with a strong affinity for self-peptides are then removed via negative selection. However, some self-reactive T cells may escape the negative selection, and constitute the potential risk of autoimmune reaction. Peripheral immune tolerance is required to limit the response of these self-reactive T cells and avoid abnormal activation of the immune system (Josefowicz et al., 2012; Klein et al., 2014). Control of self-reactive T cells through chronic antigen exposure that inactivates T cell function (T cell incompetence and T cell deficiency and differentiation of regulatory T cells (Tregs)) is required to achieve peripheral T cell tolerance (Singer et al., 2014).

DCs integrate various immune signals of the body during the induction of immune tolerance. They restore immune homeostasis by inducing apoptosis of inflammatory T cells, modulating pro- and anti-inflammatory responses, and inducing immunomodulatory function by expanding Tregs (Li et al., 2022b). Immature and tolerant DCs are able to suppress T cell activation and induce peripheral tolerance to self-antigens (Morante-Palacios et al., 2021; Yin et al., 2021).

For the treatment of diseases arising from abnormal activation, it is essential that prompt interventions are taken to maintain the dynamic balance and function of immune system. Although immunosuppressive drugs have been widely used in the treatment of autoimmune diseases and transplantation, however these drugs generally require lifelong administration and cannot cure the disease. Moreover, long term administration of immunosuppressive drugs can cause neurological, blood, renal, gastrointestinal, and immune system toxicity. Such side effects can also weaken the body’s normal immune response and increase the risk of cancer and infection (Feng et al., 2020; Montano et al., 2021). Therefore, researchers are focusing on therapies that induce immune tolerance by targeting immune cells (Ghobadinezhad et al., 2022). Those immunotherapies can establish antigen-specific immune tolerance while the rest of immune functions remains uninfluenced, alleviate symptoms, even completely cure the disease. DC based therapies are ideal immunotherapy strategies, which have shown promising results in clinical trials for diseases such as breast cancer, type 1 diabetes, multiple sclerosis, and post-transplant solid organ rejection (Phillips et al., 2019; van Pul et al., 2019; Zubizarreta et al., 2019; Que et al., 2020).

## 3 Induction of immune tolerance by DCs for disease treatment

DCs are commonly defined as specialized APCs that express major histocompatibility complex molecules and high level co-stimulatory molecules (Kushwah and Hu, 2011; Gardner et al., 2020). The main function of DCs is to capture and process exogenous antigens in peripheral tissues for presentation to T cells after migration to draining lymph nodes. DCs are able to produce great tolerance in response to environmental signals. DCs recognize a large number of pathogen-associated molecular patterns (PAMPs) and damage-associated molecular patterns (DAMPs) through pattern recognition receptors (PRRs) and toll-like receptors (TLRs) (Tiberio et al., 2018). For example, after recognizing relevant molecular patterns on bacterial and viral pathogens, DCs initiate immune response by inducing T cells and natural killer cells to clear infections (Stergioti et al., 2022).

DCs can change phenotypically and functionally in response to environmental stimuli. Based on their phenotype and function, DCs are divided into four main types (Liu, 2005; Guermonprez et al., 2019): conventional DCs (cDCs), including cDCs1 and cDCs2; plasmacytoid DCs (pDCs); monocyte-derived DCs (mo-DCs); and Langerhans cells (LCs). Different subpopulations of DCs can respond differently to environmental triggers and differentiate extensively into immunologically active helper cells, thus providing a critical link between innate and acquired immune responses (Ness et al., 2021; Li et al., 2022b). For example, cDCs1 effectively silence CD8^+^ T cells, cDCs2 promote CD4^+^ T cell proliferation, mo-DCs produce anti-tumor immunity, and LCs are widely present in skin tissues and can secrete a large number of cytokines to support the development of T cells (Devi and Anandasabapathy, 2017; Zhang et al., 2021b; Zhang et al., 2022).

DCs can also be divided into stimulated DCs (sDCs) and tol-DCs according to the characteristics of the cellular immune tolerance (Waisman et al., 2017; Passeri et al., 2021). Although there are no specific markers for tol-DCs, tol-DCs usually consist of a population of different types of immature or semi-mature DCs. These DCs are characterized by low expression of co-stimulatory molecules (CD80, CD86, and CD40), upregulation of inhibitory and regulatory receptors, and secreting high levels of anti-inflammatory cytokines and attenuate pro-inflammatory cytokine secretion (Suuring and Moreau, 2021). Presenting an antigen without activating the inflammatory effector T cell response is essential for tol-DCs to induce and maintain self-tolerance.

Exposure of DCs to drugs, such as vitamin A, vitamin D_3_, rapamycin, dexamethasone, growth factors, and cytokines (such as tumor necrosis factor and IL-10), can induce tol-DC production (Boks et al., 2012). Induction of tol-DCs has shown significant promise in alleviating autoimmune disease symptoms, improving allograft survival, and suppressing graft-versus-host disease after stem cell transplantation (Devi and Anandasabapathy, 2017; Que et al., 2020). Purification of patient-derived precursor DCs to tol-DCs for re-infusion back to patients *in vitro* has been shown to be a promising approach in clinical trials for the treatment of autoimmune diseases (Benham et al., 2015; Nikolic et al., 2020). However, the tremendous efforts and extremely high costs of isolation, purification and *in vitro* expansion of tol-DCs, and histocompatibility issues of DCs have limited the broad application of tol-DC based therapies (Chuang et al., 2022). Researchers, therefore, have focused on the *in situ* induction of tol-DCs *in vivo*.

## 4 Optimization of DC-targeted nano-delivery systems

The biomaterial composition and physicochemical properties of DC targeting nano-delivery systems such as shape, surface potential, surface modification, and loaded bioactive molecules, have a great impact on the phenotype and function of the DCs(Tkach et al., 2013; Park et al., 2015; Zhu et al., 2019; Punz et al., 2022; Uzhviyuk et al., 2022).

### 4.1 Material composition

The four major classes of materials suitable for biomedical applications are polymers, lipids, inorganic materials and proteins (Chuang et al., 2022). The constituent materials of nanoparticle carriers should be selected to ensure that they do not have toxic effects on the organism and that they have good biocompatibility and bioavailability (Horvath and Basler, 2023). For example, materials that produce strong immunostimulatory effects, such as saponin-based adjuvants and aluminum salt adjuvants, should not be selected in the face of a range of diseases in which immune system activation is predominant (Huang et al., 2020; Huis In’t Veld et al., 2022).

Polymeric nanoparticles are one of the most commonly used nanoparticle carriers for delivering therapeutic goods. Synthetic polymeric materials, such as poly (lactic acid-glycolic ester) (PLGA), poly (glutamic acid) (PGA), and nanoparticles synthesized from natural materials (such as chitosan, gelatin, and collagen), are widely studied and used in the biomedical field. For example, PLGA has been approved by the U.S. Food and Drug Administration (FDA) and the European Medicines Agency (EMA) for use in humans (Operti et al., 2021). The degradation products of PLGA are lactic and glycolic acids, and the accompanying release of degradation products has a suppressive effect on the local immune microenvironment; it also downregulates MHC-II molecules, creating immune tolerance in humans (Allen et al., 2018; Chuang et al., 2022).

Liposomes are another class of nano-delivery systems commonly used to target DCs. They are composed of phospholipids and cholesterol, also found in cell membranes, and are highly biocompatible *in vivo*. The immunological effects of DCs can be activated or inhibited by altering the surface charge, composition, hardness, and size of the liposomes during nanoparticle fabrication (Bozzuto and Molinari, 2015). For example, by adding cholesterol to lipid bilayers can enhance the stability and improve hepatic targeting of adducted liposomes (Akinc et al., 2010). Kranz et al. introduce an RNA encapsulated lipid nanoparticle (RNA-LPX), which can precisely target DCs *in vivo*. The surface charge of the nanoparticles can be modified by adjusting the amount of cationic lipids in RNA-LPX. Researchers have demonstrated that as the cationic lipid content decreased, it was shown that the site of enrichment site of RNA-LPX with neutral or slightly negatively charge shifted from the lungs to the spleen and selectively expressed in the spleen (Kranz et al., 2016).

**FIGURE 1:**
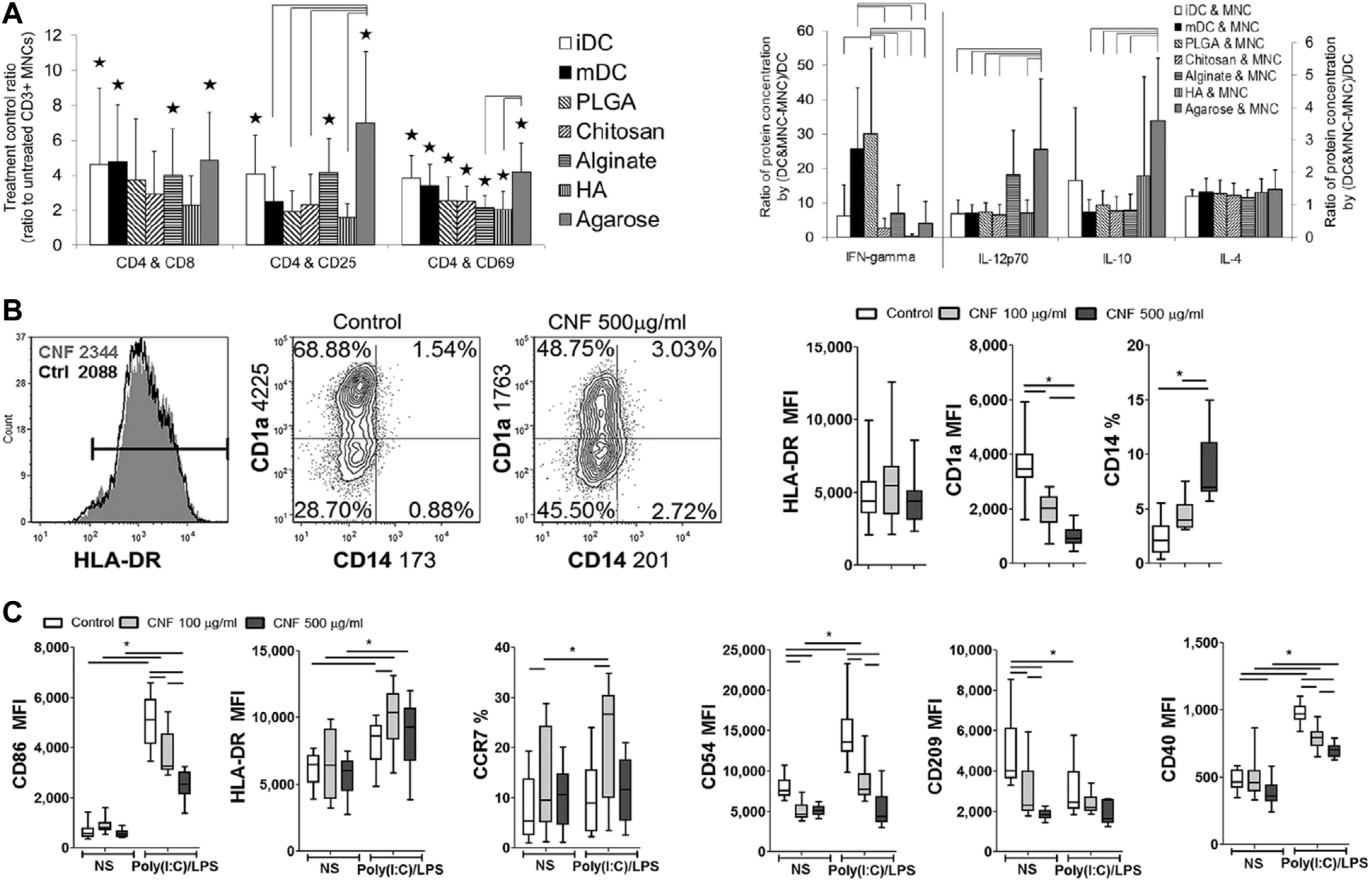
The interaction of DCs with different nanomaterials. **(A)** Multifunctional effects of DCs treated with different biomaterial films on autologous T cell-mediated phenotypes and polarization (Park et al., 2015). **(B, C)** Effects of CNFs on differentiation **(B)** and Poly (I:C)/LPS-induced maturation **(C)** of mo-DCs (Nguyen et al., 2022). Reprinted from ref (Park et al., 2015) with the permission from Wiley Periodicals, Inc., copyright 2014; Reprinted from ref (Nguyen et al., 2022) with the permission from Nature Communications, copyright 2022.

In addition to serving as a drug delivery vehicle, nanomaterials are also capable of inducing tolerogenic DCs by altering the DC phenotype. For example, agarose in the agarose gel treatment of DCs, which inhibits DC maturation and polarize T cell responses toward Th1 and Th2 and induce Treg expansion (Figure 1A) (Park et al., 2015). Cellulose nanofibers (CNFs) are able to hinder the maturation and differentiation of mo-DCs and induce human tolerant DCs; they are also able to weaken Th1 and Th17-mediated responses and induce Tregs production (Tomić et al., 2016). Cerium nanoparticles can prevent oxidative stress in DCs by reducing the level of reactive oxygen species (ROS) in DCs and reducing the level of CD86 and MHC-II expression on DCs (Figures 1B,C) (Nguyen et al., 2022). Other materials, such as gold and pSi, have little immunogenicity, do not stimulate DCs, which would otherwise lead to their activation, and are good choices for the loading of various immunosuppressive drugs (Arosio et al., 2014; Stead et al., 2018b).

By making an appropriate choice of materials, researchers can better control the safety profile of nano-delivery systems. However, this is only a single parameter that must be considered in nanoparticle design; nanoparticle size and shape also need to be taken into account.

### 4.2 Size and shape

Size of nanoparticles has a great effect on the cellular uptake of nanoparticles *in vivo*. Nanoparticle size for drug delivery in nano-delivery systems is usually controlled to be in the range of 10–1,000 nm (Sun et al., 2014). Nanoparticles can be effectively taken up by DCs through lectin-mediated endocytosis when their size is less than 100 nm. Particles larger than 200 nm are internalized by DCs through phagocytosis or by macrophagocytosis (Getts et al., 2015).

**FIGURE 2:**
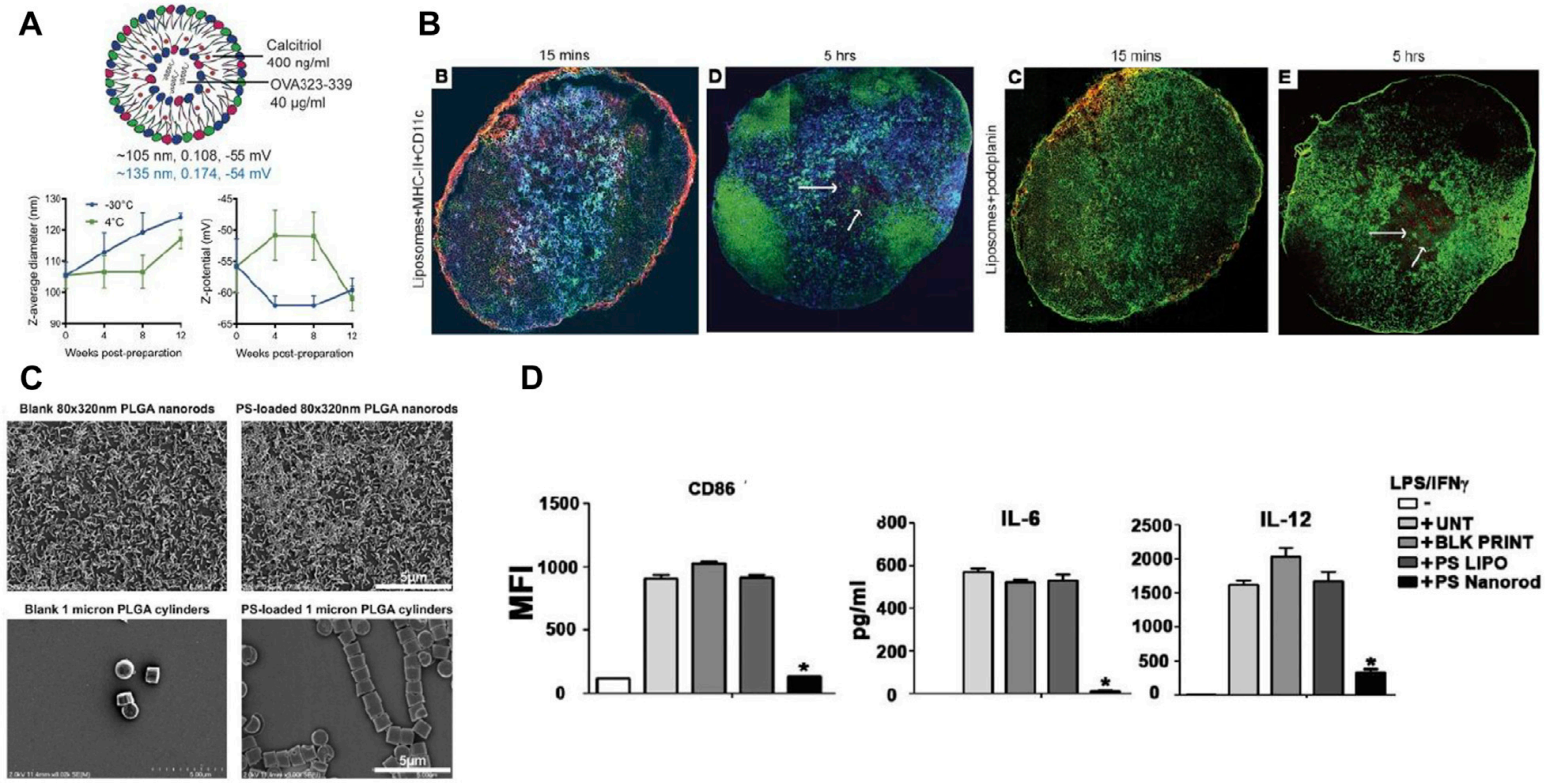
Interaction of DCs with nanoparticles of different particle size and morphology. **(A)** Size, polydispersity (PDI), and surface charge (black: thin film hydration method, blue: microfluidic method) of liposomes encapsulating calcitriol and OVA_323-339_. **(B)** Calcitriol-antigen liposomes are distributed from the injection site to the draining lymph node (dLN) after administration, subsequently into the dLNs of myeloid DCs and inflammatory mo-DCs (Galea et al., 2019). **(C)** Changing the geometry of PLGA nanoparticles by geometric manipulation of PS. **(D)** PS nanorods downregulate inflammatory responses in dendritic cells (Roberts et al., 2015). Reprinted from ref (Galea et al., 2019) with the permission from American Society for Clinical Investigation, copyright 2019; Reprinted from ref (Roberts et al., 2015) with the permission from Elsevier Ltd, copyright 2015.

Size has an impact on the *in vivo* distribution of nanoparticles. For example, Parker et al. showed that DCs readily endocytose small graphene oxide (SGO) flakes, while the plasma membrane of a macrophage readily absorbs large graphene oxide (LGO) flakes (Parker et al., 2022). Many studies have shown that nanoparticles measuring less than 200 nm can be processed by DCs in lymph nodes after injection, inducing early T-cell action (Manolova et al., 2008; Blank et al., 2013). For example, Galea et al. synthesized liposomes containing calcineurin and PD-L1 in the size range of 105–135 nm. The liposomes could target lymph nodes from the site of administration via passive drainage, causing lymph node DCs to exhibit increased PD-L1 expression (Figures 2A,B) (Galea et al., 2019). In contrast, nanoparticles larger than 200 nm will stay at the injection site or enter the spleen, liver and lymph nodes with migrating DCs after being internalized by DCs (Robinson and Thomas, 2021).

Particle size is not the only determining factor for the uptake and distribution of nanoparticles by cells *in vivo*. Nanoparticle shape is also an important character and can affect the recognize of nanoparticles by DCs, which has a great impact on the phenotype and function of DCs(Niikura et al., 2013). Rod NPs exhibits a lower internalization rate of APCs, whereas spherical NPs trigger increased phagocytosis and are more likely to accumulate in the liver, lung, and spleen (Champion and Mitragotri, 2006; Mathaes et al., 2014). By varying the shape of phosphatidylserine (PS) on nanoparticles, Roberts et al. found that rod-shaped PS-PLGA nanoparticles were more likely to induce immune tolerance than spherical PS-PLGA nanoparticles for the same particle size (Figures 2C,D) (Roberts et al., 2015).

### 4.3 Zeta-potential

Adjusting the surface charge of NPs is another key factor affecting the internalization ability, distribution, and immunogenicity of DCs. For example, highly charged nanoparticles will be more stable due to electrostatic repulsion, regardless of whether the surface charge is positive or negative in nature (Ma et al., 2011; Zhang et al., 2021a). Positively charged NPs are more strongly internalized by phagocytes rapidly through interaction with negatively charged cell membranes or through the lectin-mediated endocytosis (Li et al., 2022a). For example, modification of PLGA particles with a negative surface potential with positively charged polymers, such as polyethylene glycol (PEI) or chitosan (CS) (Wang et al., 2021), shifts the zeta potential to positive values and promotes the cellular phagocytosis of nanoparticles by interacting with negatively charged cell membranes (Zupancic et al., 2017). In addition, nanoparticles with cationic surface charge can bind anionic mRNA by electrostatic interaction and improve the transfection efficiency of mRNA to DCs (Yasar et al., 2018).

On the other side, cationic nanoparticles also have disadvantages. It has been shown that cationic liposomes generate highly electrostatic interactions with negatively charged tissues, causing nanoparticles more likely to reside at the site of administration, hindering the transport process of the nanoparticles *in vivo* (Tenchov et al., 2021; Thi et al., 2021). Moreover, cationic nanoparticles exhibit more severe cytotoxic effects compared to anionic nanoparticles. They can disrupt cell membranes, cause hemolysis and platelet deposition, and have a detrimental effect in therapeutic strategies targeting APCs to induce immune tolerance (Patra et al., 2018; Nagy et al., 2021). Studies have shown that cationic liposomes preferentially interact with negatively charged cell membranes of APCs, resulting in the activation of DCs and pro-inflammatory effects, which has negative effects in the treatment of autoimmune diseases and transplant rejection (Dangkoub et al., 2021; Nagy et al., 2021).

Negatively charged particles, although less capable of internalization, are more capable of inducing antigen-specific immune tolerance and ameliorating inflammation (Lau et al., 2022). Certain anionic preparations containing PS or 1,2-distearoylglycerol-3-phosphate glycerol (DSPG) are tolerated in mice after *in vivo* injection of bone marrow-derived DCs(Shi et al., 2007; Wu and Nakanishi, 2011). The results of Nagy et al. showed that, compared with the cationic liposomes DPTAP and DOTAP, neutral or negatively charged liposomes were efficiently absorbed by human mo-DCs and skin DCs without affecting the immunogenicity of the mo-DCs and skin DCs (Nagy et al., 2022).

### 4.4 Surface functionalization

Nanoparticles as drug delivery vehicles are readily recognized by conditioners when they are injected into the circulation. They are then engulfed by cells in the mononuclear phagocyte system and are rapidly removed from the circulation (Cao et al., 2020). However, in order to deliver sufficient quantity of systemic therapeutic agents to target tissues, these nanoparticles must remain stability and maintain long circulating time in the bloodstream. Functionalized alterations to nanoparticles can help attain this (Punz et al., 2022).

PEGylation of nanoparticles reduces nanoparticle adsorption and aggregation by serum proteins, thus hindering the clearance of nanoparticles by the mononuclear phagocyte system (Van Haute et al., 2018; Toro-Mendoza et al., 2023). PEGylation can improve the stability of liposomes, which has an effect on the cycling time and cell interaction of liposomes (Hald Albertsen et al., 2022). For example, liposome surface modification with PEG chains in a brush conformation improves the *in vivo* stealthy, prolong the circulation time (Moghimi and Szebeni, 2003; Li et al., 2021a).

PEGylation can also improve the targeting of nano-delivery systems. For example, in an LPS-stimulated mouse model of chronic inflammation, PEGylation increases the distribution and retention of nanoparticles at sites of chronic inflammation (O'Mary et al., 2017). Maleimide can react specifically and spontaneously with sulfhydryl groups on cell membranes under physiological pH conditions. This spontaneous reaction can prolong the circulation time of maleimide-functionalized nanoparticles in bloodstream and enhance the internalization of immature DCs (Lee et al., 2020). PLGA nanoparticles usually exhibit negatively charged, but by modifying positively charged materials (e.g., polyethylene glycol (PEI) and chitosan (CS)) on the surface, PLGA nanoparticles can be endowed with positive charge, enhance the phagocytosis of the nanoparticles by immune cells (Horvath and Basler, 2023). Reducing the immunogenicity of nanoparticles can be achieved by covering the nanoparticle surface with a naturally derived cell membrane (Fang et al., 2023; Sun et al., 2023).

Nanoparticle surface functionalization can also reduce the hematotoxicity and cytotoxicity of nanoparticles. For example, dendritic polymers can cause erythrocyte hemolysis, and their modification by PEG can improve the erythrocyte hemolysis response to reduce blood clotting (Santos et al., 2018). Yu et al. introduced a DC targeting nanoparticle modified with peptide antigen OVA24 and adjuvant Pam3CSK4 on the surface, which increased the negative charge on the surface, and significantly reduced the cellular toxicity to DCs(Yu et al., 2022).

**FIGURE 3:**
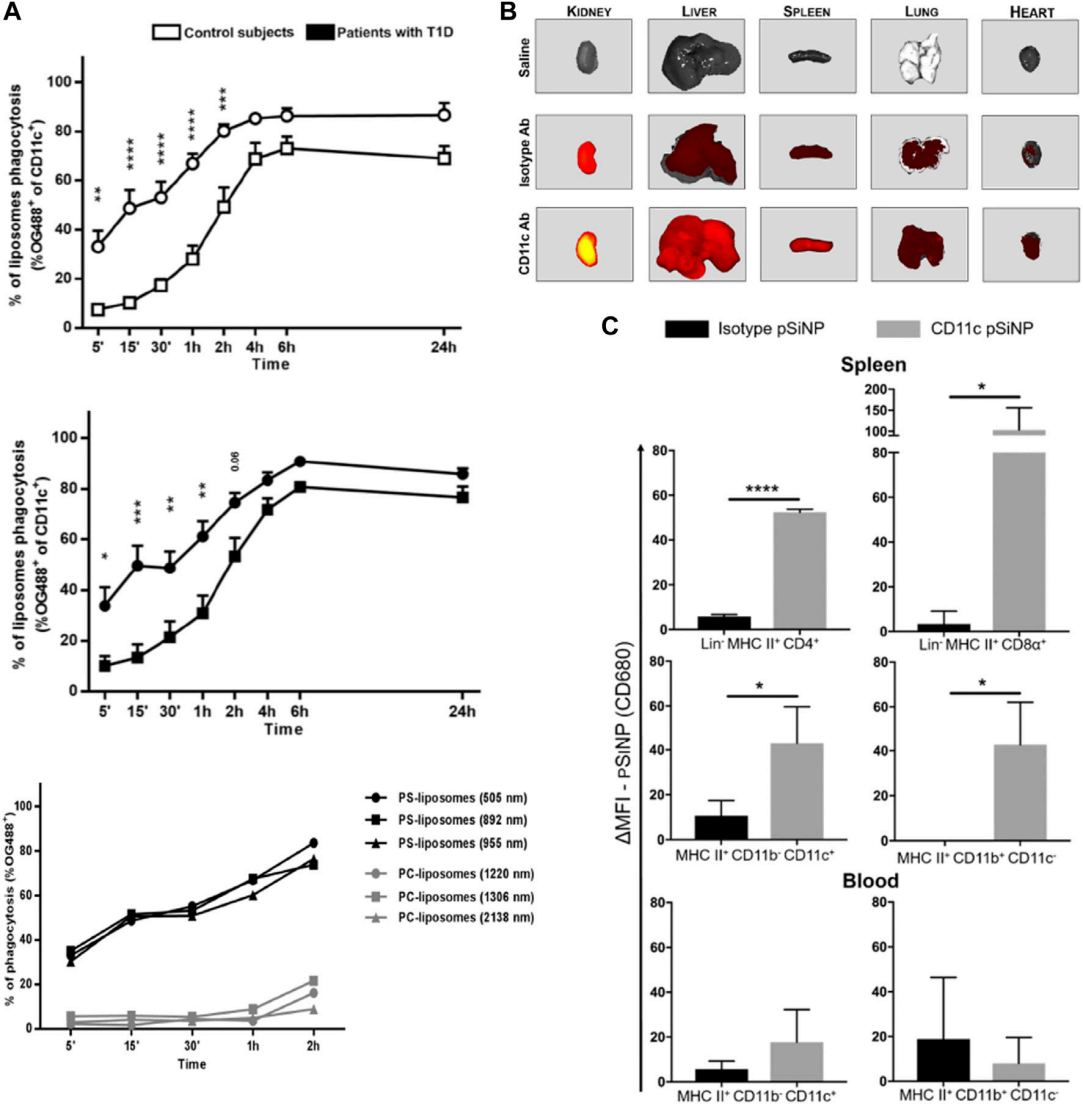
Surface modification of nanoparticles affects the interactions between nanoparticles and DCs. **(A)** The PS component in PS-liposomes is a key factor in accelerating the phagocytosis of liposomes by DCs (Rodriguez-Fernandez et al., 2018). **(B)** Modification of pSiNP with CD11c antibody enhanced pSiNP accumulation in mouse liver, lung, heart, spleen and kidney. **(C)** Modification of pSiNP with CD11c antibody increases both CD4^+^ and CD8α+ DC phagocytosis of pSiNP in spleen (Stead et al., 2018a). Reprinted from ref (Rodriguez-Fernandez et al., 2018) with the permission from Frontiers, copyright 2018; Reprinted from ref (Stead et al., 2018a) with the permission from American Chemical Society, copyright 2018.

Nanoparticles can be used as tolerogenic adjuvants through nanoparticle surface functionalization, inhibiting the maturation and differentiation of DCs. PS is a major component on apoptotic cell membranes, decorating the nanoparticle surface with PS can promote the recognition by scavenger receptors and internalization of DCs, while exerting inhibitory effects on the differentiation and maturation of DCs (Figure 3A) (Szondy et al., 2017; Rodriguez-Fernandez et al., 2018).

Modification of nanoparticles with antibodies against specific antigens present on DCs (e.g., CD11c and CD40 antibodies) and targeting agents against c-type lectins on the surfaces of DCs (e.g., mannose receptor and DCs-SIGN) enhance the targeting ability of nanoparticles against DCs and enhance the phagocytosis via receptor-mediated endocytosis (Hlavaty et al., 2015). Stead et al. found that functionalized modification of porous silicon nanoparticles (pSiNP) carrying rapamycin and OVA peptides with anti-CD11c antibodies enhanced pSiNP phagocytosis by DCs in peripheral blood and spleen; it also significantly increased Treg levels in OVA-sensitized mice (Figures 3B,C) (Stead et al., 2018a). He et al. coupled mannose on the surface of nanoparticles (OVA-PLGA NP) and significantly enhancing the OVA-PLGA NP phagocytosis of hMoDCs and promoted hMoDCs to exhibit a tolerogenic phenotype (Wen et al., 2021).

Control of the physical and chemical properties of nanoparticles enhances cargo delivery *in vivo* and the ability of nanoparticles to produce a coordinated enhancement of the therapeutic effect of the cargo in various immune activating or immune tolerant disease settings. Therefore, we next discuss the role of nanoparticles in targeting DC delivery in the context of specific diseases.

## 5 Nanodrug delivery systems targeting DCs to induce immune tolerance for disease treatment

Nanoparticles enhance the immunomodulatory effect of encapsulated cargoes. This provides nano-delivery systems with unique advantages in the delivery of therapeutic cargo to modulate immune cell activity. DC targeting nanoparticles which can induce immune tolerance are divided into four main categories depending on the therapeutic cargo they carry (Kishimoto and Maldonado, 2018; Horwitz et al., 2019): 1) Nanoparticles carrying peptides associated with autoantigens induce antigen-specific T cell production. 2) Nanoparticles carrying immunomodulatory drugs that induce the conversion of immature DCs to tolerant DCs. 3) Nanoparticles carrying nucleic acids or plasmids with gene editing effects that block the co-stimulatory signaling pathway between DCs and T cells. 4) Nanoparticles simultaneously deliver autoantigen-associated peptides, immunomodulatory drugs and nucleic acids. Here we briefly discuss the role of therapeutic cargo-targeted DCs after nano-delivery in the treatment of autoimmune diseases, allergic diseases, and transplant rejection diseases. The aggregated results are shown in Table 1.

**TABLE 1:** Summary of studies on the induction of immune tolerance by nanoparticles carrying therapeutic cargo and targeting DCs in animal models of disease.

Animal model	Material	Nanoparticle size (nm)	Zeta potential (mV)	Therapeutic cargo	Route of administration	*In vivo* results	References
EAE	Mesoporous silica	NA	NA	MOG_35-55_	IV	Disease onset and late chronic phase disease symptom reduction	Nguyen et al. (2022)
EAE	Gold particles with PEG	60	NA	ITE and MOG_35–55_	IV	Significant reduction in clinical scores	Yeste et al. (2012)
EAE	PS-liposomes	861.29 ± 130.49	−36.19 ± 5.32	MOG_40–55_	IV	Slowing down the clinical extent of disease attacks	Pujol-Autonell et al. (2017)
EAE	Liposomes	103.4 ± 28.3	−24.9 ± 9.7	ITE and MOG_35−55_	IV or SC	Significant reduction in clinical scores	Kenison et al. (2020)
EAE	Liposomes	NA	NA	m1Ψ mRNA	IV	Inhibition of disease progression	Krienke et al. (2021)
EAE	PLG	351.3 ± 21.7	14.8 ± 0.89	PLP_139-151_	IT	Significant reduction in clinical scores	Saito et al. (2020)
EAE	PLGA	114–186	NA	PHCCC	SC	Delayed onset and reduced disease severity	Gammon et al. (2015)
EAE	PLGA	582.3 ± 32	8.75	ICAM-1-binding and MOG_35-55_	IV	Prevention of EAE; significant reduction in disease clinical scores	Wang et al. (2022a)
EAE	PLGA	378.4	−34.8	MOG_35-55_ conjugated with glucosamine or MOG_35-55_ mixed with mannose	IV	Significant reduction in clinical scores	Triantafyllakou et al. (2022)
T1D	RGD- and mannose-modified chitosan	322.5 ± 6.1	34.9 ± 0.5	Heart shock protein 65–6×P277	oral	Prevention of diabetes in NOD mice	Chen et al. (2018)
T1D	Gold particles with PEG	60	NA	Β-cell antigen proinsulin and ITE	IP	Stopping the development of T1D	Yeste et al. (2016)
T1D	PEG-PLGA and cationic lipid	138	23	2.5mi peptide, Cas9 mRNA, CD40 gRNA, CD80 gRNA an CD86 gRNA	IV	Suppressed pancreatitis and inflammation and prevented the eventual development of T1D	Luo et al. (2020)
TrpHEL	PLGA and PLA-PEG	100	NA	HEL_46-61_ and rapamycin	IV	Improving vitiligo symptoms	Zhang et al. (2021c)
FA	mPEG-PDLLA	175.5 ± 6.5	2.81	R848 and OVA_33-47_	oral	Prevention of allergic reactions	Hong et al. (2019)
AR	mPEG-PDLLA and NGRPEG-PDLLA	17.83 ± 0.28	−6.01 ± 0.96	Xanthatin	nasal administration	Suppression of AR recurrence	Zheng et al. (2020)
murine and NHP model	pSi	<200	NA	Rapamycin and OVA_323−339_	IV	DC-targeted effects in mouse and NHP models; OVA sensitized mice Treg elevation	Stead et al. (2018a)
skin allografts	PEG-PLGA	100	25	CD40 siRNA	IV	Significantly prolonged graft survival time	Wang et al. (2022b)
skin allografts	PEG-PLGA and cationic lipid	100	10	Cas9 mRNA and CD40 gRNA	IV	Relieves graft rejection and prolongs graft survival time	Zhang et al. (2019)
skin allografts	PEG-bl-PPS	39.3 ± 1.5	NA	Rapamycin or tacrolimus	intradermal injection	Extended survival time of grafts	Dane et al. (2011)

### 5.1 Induction of autoantigen-based immune tolerance by DC targeting nanoparticles

Antigen-specific therapeutic strategies have been extensively studied for diseases linked to predominantly activated immune systems. In such therapies, DCs with tolerogenic phenotypes are utilized for antigen presentation to induce antigen-specific immune tolerance for autoimmune diseases (Pozsgay et al., 2017; Ness et al., 2021). These therapies do not require reducing inflammatory signaling by modulating cellular signaling pathways or preventing cells from overproducing antibodies or migrating to disease sites, disease associated autologous lymphocyte activity can be attenuated by modulating existing cell functions and induce antigen-specific immune tolerance (Castenmiller et al., 2021; Passeri et al., 2021).

Antigen-specific therapies focus on the immune cells and autoantigens involved in the onset of disease symptoms. These therapies were initially used in the prevention and treatment of autoimmune diseases due to the presence of multiple different and recognizable antigens, and have been subsequently applied in the treatment of allergic diseases and transplant rejection. By using nanoparticles carrying peptides derived from their own antigens, the biological instability and poor pharmacokinetics of free peptides or proteins into the body can be modified. Moreover, nanoparticles are capable of improving the delivery efficiency of peptides and avoiding *in vivo* degradation of peptides (Asadirad et al., 2019; Yang et al., 2023). DC targeting nanoparticles carrying autoantigens can induce DCs with tolerance phenotypes. When the antigens are digested by DCs, the antigenic peptide fragments are expressed on the surface of the DCs and presented to T cells via the MHC-TCR pathway (Waeckerle-Men and Groettrup, 2005; Shen et al., 2006).

Apoptotic cell mimicking PS-liposomes can be recognized and phagocytosed by DCs. Pujol-Autonell et al. significantly reduced the expression of CD86, CD40, and MHC class II molecules after co-culturing DCs with PS-liposomes loaded with MOG_40-55_. EAE mice were administered with PSMOG-liposomes, PS-liposomes, and MOG peptide alone separately, but PS-liposomes showed no therapeutic effect. PSMOG-liposomes, however, produced a significant reduction in disease clinical scores and demonstrated better therapeutic effect than MOG peptide alone (Pujol-Autonell et al., 2017). Nanoparticle-encapsulated autoantigen peptides have been applied in the treatment of other autoimmune diseases. By replacing the nanoparticle-encapsulated autoantigens, nanoparticles can be applied to the treatment of other autoimmune diseases. For example, they replaced the MOG peptide encapsulated in PS-liposomes with insulin peptide, this PS-liposomes could promote tolerogenic features on DCs in T1D (Rodriguez-Fernandez et al., 2018).

Mannose-modified nanoparticles are also often used for the targeted delivery of autoantigens to DCs. Chen et al. used chitosan nanoparticles modified with mannose and peptide arginine to carry H6P antigen and prevent the onset of diabetes in NOD mice via oral administration and induced antigen-specific T-cell tolerance (Chen et al., 2018). He et al. used mannose-modified PLGA nanoparticles loaded with OVA (OVA-mann-PLGA NP), co-incubating them with hMoDCs, and found that the hMoDCs showed increased production of pro-inflammatory cytokines IL-10 and TNF-α and decreased production of anti-inflammatory cytokine IL-6. When mice were immunized with OVA-mann-PLGA NP, OVA-sensitized mice exhibited immune tolerance to OVA allergens (Wen et al., 2021).

Delivery of specific peptides using nanoparticles can block signaling pathways on DCs. The NFAT signaling pathway in DCs is responsible for inducing effective T cell activation and graft rejection. Blocking the NFAT signaling pathway on DCs can inhibit the proliferation of antigen-specific T cells and plays a role in inducing graft tolerance (Otsuka et al., 2021; Colombo et al., 2022). Colombo et al. screened for VIVIT peptides with a high affinity for calcium-regulated neurophosphatase (CN), using them to inhibit the interaction between CN and NFAT to and block the CN/NFAT pathway. Delivery of VIVIT peptides using nanoparticles targeting DCs not only prevents graft rejection during treatment but also induces long-term skin graft tolerance after the end of treatment compared to the immunosuppressant FK-506 in a skin graft model (Colombo et al., 2022).

In the inflammatory setting of disease, although some therapeutic benefit can be achieved by delivering autoantigens, co-delivery with other tolerance agents may achieve better efficiency by ensuring DCs receive all signals while inducing optimal tolerance. For example, co-delivery of therapeutic cargoes via nanoparticles, such as immunosuppressive drugs, adjuvants, cytokines, vitamin D_3_, and RNA with antigens can enhance the ability of nanoparticles to induce tolerance phenotypes in DCs and achieve better therapeutic effect on those diseases (Casey et al., 2018; Hong et al., 2019; Jung et al., 2019; Liu et al., 2022).

### 5.2 Delivery of immunomodulators to induce tol-DCs for disease treatment

Immunomodulators are delivered using nano-delivery systems, which include: 1) Immunosuppressive drugs. 2) Aryl hydrocarbon receptor (AhR) ligand agonists. 3) Glutamate metabotropic receptor-4 agonists. 4) Anti-inflammatory factors (Horwitz et al., 2019; Lamendour et al., 2020). Through the effect of these immunomodulators on DCs it is possible to transform immature DCs into DCs with a tolerogenic phenotype. This therapeutic approach is a promising strategy for establishing permanent specific immune tolerance, it shows significant promise in suppressing autoimmune diseases, in prolonging the survival of allografts, and in the treatment of allergic diseases (Feng et al., 2019; Que et al., 2020). However, many immunosuppressive agents, such as methotrexate, rapamycin, and dexamethasone, have limited biological activity *in vivo*, are randomly and widely distributed in the body, and cause damage to the liver, kidneys, and gastrointestinal tract after systemic administration. The delivery of immunomodulators via nanoparticles not only reduces drug toxicity, but also improves the targeting of drug release. This increases the therapeutic effect and safety of the drug while reducing the drug dose and toxicity (Hong and Dobrovolskaia, 2019; Li et al., 2021b).

Rapamycin is a macrolide antibiotic with immunosuppressive activity and acts as an inhibitor to block the mammalian rapamycin (mTOR) pathway. Rapamycin can reduce the expression of co-stimulatory markers on DC surfaces, prevent complete T cell activation, and promote Tregs expansion (Raich-Regue et al., 2015; Herrero-Sanchez et al., 2016). Delivery of rapamycin using nanoparticles significantly reduces surface co-stimulatory molecule expression and inhibits the maturation of DCs compared to free rapamycin (Haddadi et al., 2008). Co-delivery of rapamycin with disease-associated antigens can induce antigen-specific Treg generation along with the induction of tolerogenic DCs. Kishimoto et al. used rapamycin-loaded PLGA nanoparticles (SVP-Rapamycin) co-administered with antigen and found that they could greatly reduce the corresponding antibody levels and induce tol-DCs and durable immune tolerance effects (Kishimoto et al., 2016). This was demonstrated by the isolated DCs from animals treated with antigen and SVP-Rapamycin co-administration, which could suppress the proliferation of antigen-associated T cells, while enhance Treg differentiation. The combined delivery of rapamycin and antigen by nanoparticles has also shown therapeutic efficacy in animal disease models. For example, PLGA nanoparticles encapsulated with rapamycin and peptide antigen, which can stimulate the induction of tolerogenic DCs, promote the generation of Tregs, and induce antigen-specific tolerance in models, such as EAE (Maldonado et al., 2015). Zhang et al. found that BMDCs treated with nanoparticles containing rapamycin and NPHEL_46-61_ (NPHEL_46-61_/Rapa) could inhibit CD4^+^ T cell proliferation while inducing Treg differentiation. Treatment of vitiligo mice with NPHEL_46-61_/Rapa enhanced IL-10 expression and inhibited IFN-γ and IL-6 expression in diseased mice (Zhang et al., 2021c).

Drugs with anti-inflammatory and immunosuppressive effects are also often encapsulated in nanoparticles as therapeutic cargoes for the targeting delivery towards DCs. Zheng et al. used polymeric micelles to target CD13 receptors on the surfaces of DCs; the micelles acted as vehicles to deliver Xanthium (NGR-XT-PM) (Zheng et al., 2020). Compared with XT-PM and free XT, NGR-XT-PM considerably reduced the expression of CD80, CD86, and I-A/I-E molecules on the surfaces of DCs. In a mouse model of allergic rhinitis NGR-XT-PM inhibited the recurrence of allergic rhinitis, while relieving nasal symptoms; its therapeutic effect was superior to that of free XT and the commercial product Budesonide. Kim et al. used PLGA nanoparticles to carry both dexamethasone and OVA, and NP [OVA + Dex] treatment caused immature DCs to be converted to tolerant DCs. Moreover, OVA-specific immune tolerance was induced in mice by oral or intravenous administration (Kim et al., 2019b).

The AhR is a ligand-dependent transcription factor receptor and plays an important role in the control of immune responses. Regulation of the function of antigen-presenting cells, such as DCs and macrophages, can be achieved through modulation of AhR signaling, which can impact T-cell differentiation (Gutierrez-Vazquez and Quintana, 2018). ITE is a potent AhR agonist that binds directly to the AhR. Activation of the AhR can lead to the inhibition of co-stimulatory molecule expression and cytokine secretion of DCs, induction of a tolerogenic phenotype in DCs, and inhibit the immune response (Quintana et al., 2010). Yeste et al. delivered both ITE and β cell antigen proinsulin to DCs in nonobese diabetic mice using nanoparticles, preventing the development of type 1 diabetes and inhibiting NF-kB signaling in DCs through a SOCS2-dependent mechanism. This reduced cell surface CD40 and CD86 expression and induced a tolerogenic phenotype in DCs (Yeste et al., 2016). Kenisona et al. demonstrated that NLPITE + MOG treatment suppressed EAE onset symptoms and induced long-term immune tolerance in an EAE model while increased the number of MOG-specific IL-10^+^ CD4^+^ T cells was detected in the central nervous system of NLPITE + MOG-treated EAE mice (Kenison et al., 2020).

The inflammatory status and phenotype of tolerance of DCs can be regulated using metabolic modulators. For example, glutamine is expressed at high levels during organismal inflammation, and pDCs and cDCs expressed high level of its receptor mGluR4. The release of glutamate from DCs during inflammation facilitates Treg production by activating mGluR4 signaling on DCs (Julio-Pieper et al., 2011). The use of agonists of mGluR4, such as Cinnabarinic acid and PHCCC, can induce Treg production, enhance immune tolerance, and suppress neuroinflammation in the treatment of autoimmune diseases (Fazio et al., 2014; Peferoen et al., 2014). The application of nanoparticles to deliver agonists of mGluR4 has not been well studied. Jewell et al. used PLGA nanocarriers and liposomes to deliver PHCCC, to investigate the effect of controlling immune cell metabolism on immune tolerance in the body and to validate it with autoimmune disease models (Gammon et al., 2015; Gammon et al., 2017). Their results demonstrated that delivery of PHCCC using nanocarriers significantly reduced the toxicity of PHCCC using soluble PHCCC as a comparison. DCs can effectively internalize PHCCC NP, and this internalization has a dose dependence. Co-incubation of PHCCC NP with LPS-stimulated DCs revealed that PHCCC NP significantly reduces CD40, CD80, and CD86 expression on activated DCs, and inhibits T cell proliferation, increases Treg expansion, and decreases IFN-γ secretion. In EAE mice treatment experiments, PHCCC NP delayed the onset and reduced the severity of EAE compared to soluble PHCCC.

1,25-Dihydroxyvitamin D3 (aVD3) can exert immunomodulatory and anti-inflammatory effects by controlling different DNA methylation modifications in the metabolic and immune pathways of DCs to induce stable and reproducible tol-DCs (Lamendour et al., 2020; Gallo et al., 2023). Jung et al. synthesized nanoparticles that could deliver both aVD3 and OVA, and NP(OVA + aVD3)-treated DCs exhibited reduced expression of MHC II molecules, CD80, and CD86, and low secretion of pro-inflammatory cytokines IL-1β, IL-6, IL-12, and TNF-α. Tolerogenic DCs induced by NP(OVA + aVD3) can effectively induce Treg differentiation. Oral or intravenous administration of NP(OVA + aVD3) to mice can lead to OVA-specific tolerance (Jung et al., 2019).

### 5.3 Targeting DCs with gene disruption technology for disease treatment

In addition to the induction of tolerance in DCs through the delivery of disease-associated antigens and immunomodulators, and thus the induction of immune tolerance in the body, another promising approach to modulating the immune system is the direct regulation of the expression of co-stimulatory molecules on DCs through the delivery of nucleic acids.

Effective T-cell activation relies on two conditions being met: 1) Antigen-specific TCR binding with MHC molecules on APCs (Zhang and Vignali, 2016). 2) The co-stimulatory effect of the co-stimulatory receptors on T cells and their corresponding ligands on antigen-presenting cells (Bluestone et al., 2015). Simultaneous engagement of costimulatory molecules between T cells and APCs when the co-stimulatory pathway is blocked, T cells lose their effector function and become “incompetent” unable to activate efficiently, either by differentiating into Tregs to induce immune tolerance or by being instructed to undergo apoptosis as a result of clonal clearance (Bluestone et al., 2015). In view of this, targeted manipulation of co-stimulatory pathways on DCs and T cells can alter the T cell activation status and thus induce immune tolerance. This is a feasible approach to the induction of autoimmune tolerance and transplantation tolerance, as well as to the treatment of allergic diseases (Gu et al., 2006; Suzuki et al., 2010; Zheng et al., 2010).

Nucleic acids used to regulate the immune system include plasmid DNA (pDNA), messenger RNA (mRNA), small interfering RNA (siRNA), and microRNA. When nucleic acids administered directly, they are readily degraded by nucleases *in vivo*, and also potentially lead to toxic reactions due to the presence of an anionic phosphate backbones and off-target effects (Janas et al., 2018; Kim et al., 2019a). The challenges inherent to the *in vivo* delivery of nucleic acids, however, can be addressed using carriers composed of lipids, polymers, and inorganic materials. This produces cellular immunity through *in vivo in situ* cell reprogramming (Zhang et al., 2021d; Bogaert et al., 2022; Lam et al., 2023). For example, specific delivery of anti-sense oligonucleotides targeting CD80, CD86, and CD40 to DCs can inhibit the expression of co-stimulatory molecules on DCs and treat autoimmune diseases by inducing Treg (Machen et al., 2004; Phillips et al., 2008; Engman et al., 2015).

**FIGURE 4:**
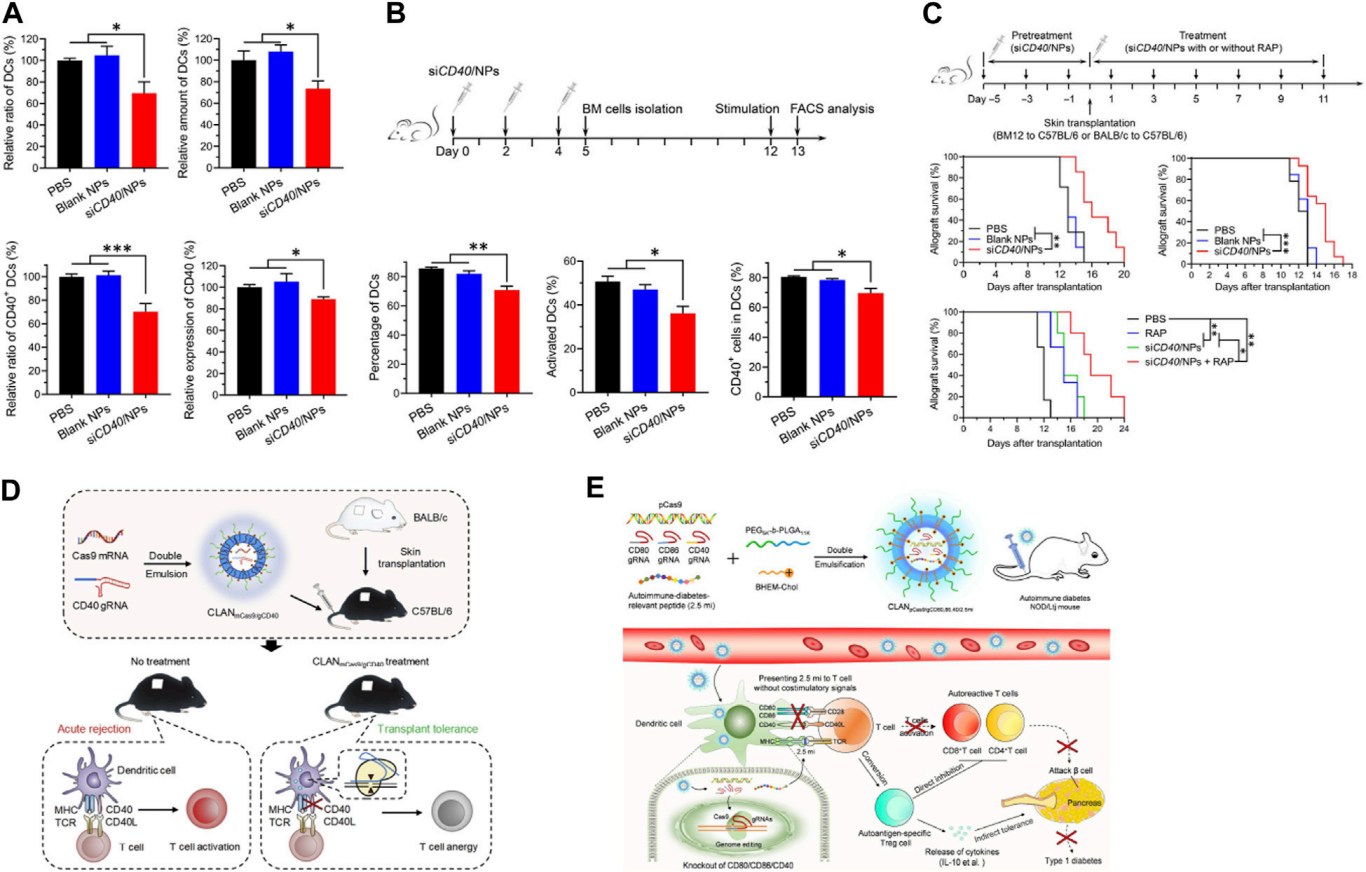
Nanoparticle delivery of nucleic acid cargo can inhibit the expression of co-stimulatory molecules in DCs and restore immune tolerance. **(A)** siCD40/NPs hinder the differentiation and downregulate CD40 expression of DCs *in vivo*. **(B)** siCD40/NPs hinder the differentiation and maturation of BMDCs *ex vivo*. **(C)** Significant prolongation of skin allograft survival by siCD40/NPs (Wang et al., 2022b). **(D)** Reprograming DCs using CLANmCas9/gCD40 to induce transplantation tolerance (Zhang et al., 2019). **(E)** Restoration of autoantigen-specific tolerance by an all-in-one nanomedicine consisting of CRISPR-Cas9 and the 2.5mi peptide (Luo et al., 2020). Reprinted from ref (Wang et al., 2022b) with the permission from American Association for the Advancement of Science’s, copyright 2022; Reprinted from ref (Zhang et al., 2019) with the permission from Elsevier Ltd, copyright 2019; Reprinted from ref (Luo et al., 2020) with the permission from American Chemical Society, copyright 2020.

Among the nucleic acid-based therapeutic approaches, RNA-based therapies have unique advantages. Unlike DNA has to be delivered into the nucleus of the target cell for proper expression (Fu et al., 2020), RNA can easily be functional in the cytoplasm and exhibits a higher therapy efficiency, therefore is preferred as the therapeutic cargo for nano-delivery. Wang et al. developed a PLGA-based siRNA nanoparticle delivery system (siCD40/NPs), using this system, they successfully delivered CD40 siRNA into hematopoietic stem cells and myeloid progenitor cells of DCs. By down-regulating the expression CD40, siCD40/NPs could inhibit the differentiation and maturation of DCs, suppress alloimmune response, prolong the skin graft survival in mouse allogeneic skin transplantation (Figures 4A–C) (Wang et al., 2022b). Wang et al. introduced a Cas9 mRNA (mCas9) and guide RNA targeting CD40 (gCD40) simultaneous encapsulating PLGA-based cationic lipid-assisted nanoparticles (CLAN). CLANmCas9/gCD40 not only effectively delivered mCas9 and gCD40 to DCs, but also significantly reduced the CD40 expression on the DCs, leading to the expansion of Tregs. CLANmCas9/gCD40 significantly inhibited the graft rejection, and prolonged the skin graft survival in a skin graft rejection model (Figure 4D) (Zhang et al., 2019). Wang et al. also developed an autoimmune associated peptide (2.5mi), CRISPR-Cas9 plasmid (pCas9), gCD40, gCD80, and gCD86 simultaneous encapsulating CLAN (CLANpCas9/gCD80,86,40/2.5mi). CLANpCas9/gCD80,86,40/2.5mi could knocked out the co-stimulatory molecules CD80, CD86 and CD40 on DCs after targeting delivery to DCs, induced the generation of tolerant phenotypes in DCs, triggered the expansion of 2.5mi peptide-specific Tregs, and effectively prevented the development of T1D in mice (Figure 4E) (Luo et al., 2020).

### 5.4 Administration routes of DC targeting nanoparticles

The distribution and immunological effects of nanoparticles *in vivo* are influenced by different drug delivery routes (Casey et al., 2018; Ackun-Farmmer and Jewell, 2023). Therefore, when delivering nanoparticles for disease treatment, it is equally important to select the appropriate therapeutic cargo as well as the suitable drug delivery route.

Lymphocytes residing in the spleen, liver, and lymph nodes are commonly employed to induce immune tolerance. Nanoparticles loaded with immunomodulation drugs can be administered intravenously (IV), subcutaneously (SC), and intraperitoneally (Dangkoub et al., 2021). IV administration is the most commonly chosen routes of nanoparticles administration, nanoparticles enter the circulation directly after administration, where they are almost entirely bioavailable in the bloodstream (Ackun-Farmmer and Jewell, 2023). After IV administration, the nanoparticles can distribute and internalized by the APCs in the liver and spleens. However, after IV administration, nanoparticles can also accumulate in non-specific tissues and result in unwanted side effects. The nanoparticles may also be degraded by enzymes and cleared by the liver, which can lead to decreased drug utilization (Li et al., 2022a; Casey et al., 2022). Krienke et al. described a liposome-based antigen encoded mRNA delivery system (mRNA-LPX). After IV administration, mRNA-LPX was able to be internalized by lymphoid tissue resident CD11c^+^ APCs throughout the body, leading to successful expression of antigen mRNA in DCs as well as the expansion of exhausted antigen specific T cells, which induced antigen-specific immune tolerance and demonstrated promising therapeutic efficacy in mice models of EAE (Krienke et al., 2021). Compared to IV administration, intradermal and subcutaneous injection enable targeting of APCs in skin and draining lymph nodes, such as Langerhans cells and DCs (Deckers et al., 2018). Dul et al. conjugated PI_C19-A3_ peptide to AuNPs, and AuNPs was intradermal administrated using microneedles (MNs). After administration, AuNPs was uptaken by epidermal LCs and dermal DCs of skin tissue. AuNPs was able to inhibit the activation of mo-DCs after co-culture, and those mo-DCs were capable of presenting antigen peptide and promote the expansion of antigen specific T cells. Next, AuNPs will be further validated as a potential therapeutic tool in the clinic (Dul et al., 2019).

In addition to invasive administration methods, non-invasive administration methods (e.g., oral routes, intranasal routes, endotracheal routes, etc.) have the advantages of reducing the difficulty of administration, improving patient compliance, and reducing the burden on patients. Through the oral route nanoparticles can reach gut-associated lymphoid tissues and encounter DCs in these tissues to induce immune tolerance (Chen et al., 2018; Li et al., 2023). It should also be noted that after oral administration of nanoparticles, the amount of drug entering the circulation tend to be reduced due to first pass elimination, and the high acid environment in the stomach may result in the degradation of nanoparticles (Abeer et al., 2020; Horvath and Basler, 2023). Studies have demonstrated that less than 10% of the total amount of drug administered could enter the circulation after oral administration of nanoparticles (Ren et al., 2023). Intestinal administration can also deliver nanoparticles into the spleen and achieve targeting of DCs. Yeste et al. developed NPs containing the AhR ligand ITE and MOG_35-55_(NP_ITE+MOG_). After i. p. administration, NP_ITE+MOG_ was capable of inducing tolerogenic DCs and expansion of Tregs, reducing Th1 and Th17 cytokine production, suppressing the development of EAE, an experimental model of MS (Yeste et al., 2012). Intratracheal administration is also an effective method of administration for the induction of immune tolerance by nanoparticles. Saito et al. synthesized poly (lactide-co-glycolide) (PLG) nanoparticles carrying myelin proteolipid protein fragment (PLP_139-151_) (Nano-PLP), and compared the therapeutic efficacy of Nano-PLP administrated via intravenous and intratracheal administration in a mouse model of EAE. They interestingly found that compared to IV injection, intratracheal administration significantly improved the utilization of Nano-PLP (Saito et al., 2020).

## 6 Summary and prospects

With the ongoing research into the mechanisms underlying autoimmune diseases, rejection, and allergic diseases, many methods targeting the induction of tol-DCs have been developed and evaluated in preclinical trials. Most clinical trials for tol-DC induction utilize *in vitro* isolation and purification of DCs from the patient’s own sources, which are prepared as tol-DC vaccines and then infused back into the patient (Passeri et al., 2021). Although many clinical trials have demonstrated that the induction of tol-DCs is a viable method for the treatment of autoimmune diseases, rejection, and allergic diseases, the difficulty and high cost of operation limits the widespread use of this technology. In addition, after *in vivo* administration of tol-DCs vaccine, exogenous semi-mature DCs may be transformed from a tolerant phenotype to activated DCs with the ability to activate T cells after inflammatory stimuli, and promoting the immune response instead. Therefore, how to maintain the immunomodulatory effects of tol-DCs in an abnormal, inflammatory environment, *in vivo*, is a key challenge.

DC targeting nano-delivery system is a promising strategy for programming *in situ* DCs *in vivo*. Although a large number of fundamental studies have demonstrated the therapeutic effects of DC-targeted nanoparticles in autoimmune diseases, transplantation, and allergic diseases, nanodrugs entered clinical trials to date were developed for oncology therapy. Clinical researches for the development of DC-targeted nanomedicines focusing on autoimmune diseases and organ transplantation are still limited, and challenges remain for the clinical translation of nanoparticles induced tolerant *in situ* DCs *in vivo*.

Despite the great potential of nanoparticles in the induction of tol-DCs, the DC targeting nanomedicine-based therapy development still face plenty of difficulties: the difference of immune system and genetic environment between animal models and human bodies, optimal targets for the diseases, optimal administration strategies for the nanomedicine, biosafety evaluation of nanoparticles, and *in vivo* monitoring of nanomedicine induced tol-DCs. The solutions for these problems still require a lot of sustained efforts in basic and clinical research.
